# Study of individual erythrocyte deformability susceptibility to INFeD and ethanol using a microfluidic chip

**DOI:** 10.1038/srep22929

**Published:** 2016-03-11

**Authors:** Lihong Liu, Sha Huang, Xiaoying Xu, Jongyoon Han

**Affiliations:** 1 School of Pharmaceutical Sciences, Southern Medical University, Guangzhou, 510515, China; 2 Department of Electrical Engineering and Computer Science, Massachusetts Institute of Technology, 77 Massachusetts Avenue, Cambridge, MA 02139, USA; 3 Department of Biological Engineering, Massachusetts Institute of Technology, 77 Massachusetts Avenue, Cambridge, MA 02139, USA

## Abstract

Human red blood cells (RBCs) deformability *in vitro* was assessed during iron dextran (INFeD) loading and/or ethanol co-administration using microfluidic deformability screening. The results showed donor-specific variations in dose dependent deformability shift were revealed below 500 μg/mL iron dextran. Two out of nine blood samples exhibited significant cell stiffening at 500 μg/mL iron dextran loading concentration (p < 0.05, Tukey test). More interestingly, co-administration of moderate amount of ethanol was identified to have significant protective effects on RBC deformability. We also noted that ethanol can reverse the deformability of impaired RBCs. Meanwhile obvious donor dependent response to ethanol administration on RBC deformability was noted using our biomimetic microfluidic chip.

The ability of RBCs to deform plays an important role for effective blood microcirculation. The biconcave shape of RBCs (8-μm-diameter) is well known to allow RBCs to have a high flexibility and to pass through small blood capillaries and thin slits of splenic sinusoids. Compromised RBC deformability could induce risks relating to blood blockage or excessive mechanical clearance of RBC in spleen^1^. Given the important role of RBC deformability, several approaches have been used in the past to assess RBC deformability as follows. Previously, the deformability of the healthy and iron deficient blood samples were compared by passing RBCs through 5 μm filters^2^, or determining RBC elongation index (EI) using a laser diffraction viscometer (i.e. ektacytometer)^3^,^4^. However, most of these clinical studies relied on data from completely different patient pools, failing to address the notably inherent inter-individual variability from different blood donors. Furthermore, these measurements revealed only bulk RBC deformability, failing to provide single-cell level information. Ability to monitor minor population of RBCs with abnormal deformability is important, since lysis/rupture of these RBCs can be the source of downstream complications.

Parenteral iron dextran (INFeD) has been used to iron stores replete in patients on chronic hemodialysis, with chronic gastrointestinal losses, and in surgical patients refusing blood transfusion. However 26% of patients experience side-effects, most of them all are mild, but 3% patients have more severe symptoms^5^. Despite adverse reactions, INFeD is still an effective iron repletion therapy that is widely used in patients with iron-deficiency anemia. Therefore INFeD is chosen as model drug in our study. The mechanism(s) leading to INFeD-induced symptoms remains unclear but is probably multifactorial. Hamstra *et al.* found that severe reactions were associated with infusion of larger iron doses (more than 500 mg (iron, ~100 μg/mL))^6^. However, patients with chronic kidney disease generally received INFeD at a median dose of 1000 mg (iron, ~200 μg/mL))^7^ indicating that side-effects were more prevalent in young women, especially those with pre-existing collagen vascular disease^5^. Hypersensitivity to dextran or direct toxic effects of iron were implicated as the likely causes of adverse reactions to INFeD in patients with collagen vascular disease^6^.

Although extensive research has been carried out exploring the biochemical effect on RBCs, to the best of our knowledge, the patient-specific effect of INFeD on single RBC deformability remain under-explored. From the perspective of RBC deformability, ethanol exhibits a dose-dependent biphasic effect^8^. However, it is also unclear whether moderate ethanol consumption could protect individuals from INFeD induced injury through improving RBC deformability.

A rapid blood deformability assay that assesses the differential INFeD induced deformability response on the same individual is therefore highly desirable. Therefore, we propose to study INFeD induced injury and ethanol protection on RBC mechanical deformability by a simple microfluidic platform (Fig. 1) which mimics RBC microcirculation in real blood capillary and captures hundreds of RBCs in a few minutes. Our chip involves periodically spaced, triangle-shaped pillars, and the constrictions in series along the length of the channel enable repeated measurements of the same cell, increasing precision. RBC deformability is dynamically quantified by measuring the average transit velocity of single red cell passing the repeated constrictions. The higher transit velocity corresponds to better deformability. Detailed fabrication steps were described by Bow *et al.*^9^. Due to diverse inter-individual susceptibility to INFeD and ethanol loading (Fig. 1), the same prescribed dose could lead to different effects on different individuals^10^. The same device has previously used for monitoring malaria infected RBC stiffening^11^, blood storage legion^12^ and spleen filterability of RBCs^13^.

## Results

We investigated that the effect of INFeD loading on RBC deformability (Fig. 2). Fresh blood from nine healthy subjects (subject A–I, Fig. 2) were treated with 0.5–5000 μg/mL of INFeD solution *in vitro* and the deformability of single RBCs was characterized by their transit velocity when passing through the microfluidic deformability cytometer^11^. At the highest INFeD loading concentration (5000 μg/mL), significant RBC stiffening was observed for all blood samples (p < 0.05, Tukey test). Two out of nine blood samples exhibited significant cell stiffening at lower INFeD loading concentration (500 μg/mL, p < 0.05, Tukey test). One blood sample showed significant stiffening even at the lowest INFeD concentration (Subject D, Fig.2, p < 0.05, Tukey test).

Given that ethanol displays a dose dependent biphasic effect on normal RBC deformability^14^, we also investigated whether the damaging effect of INFeD overloading on RBC deformability could be reduced or tapered with carefully controlled ethanol co-administration. The results showed co-administration of small amount of ethanol is found to improve RBC deformability (Fig. 3), morphology (Fig. 4b,c) against INFeD overloading. And our data suggests an interesting dose-dependent protective effect of ethanol on RBCs from INFeD induced injury. Ethanol was co-administrated at 0.03–3% v/v. In two out of six samples, a significant increase in RBC deformability was detected when 0.03% of ethanol was co-administrated with INFeD (Fig. 3, subject L and N). In two other subjects, increase in RBC deformability only occurred at slightly higher ethanol concentration of 0.3% (Fig. 3, subject J and M), whereas in both subject K and O, an increasing trend seemed to appear after 0.03–0.3% of ethanol co-treatment, but not statistical significance can be concluded (Fig. 3, subject K and O, p > 0.05, Tukey test).

Fig. 4 illustrates how INFeD-treated RBC morphology can change with varied ethanol concentrations: whereas stomatocytes are typically formed with INFeD treatment alone (Fig. 4a), low concentration of ethanol co-administration (<0.3% v/v) could preserve the discoid shape of normal RBCs to a large extent (Fig. 4b,c). However significant echinocytosis was observed among over 90% of RBC populations when 3% ethanol was added (Fig. 4d). The improvement or deterioration of RBC morphology also was observed in parallel with whole cell deformability. However, too much ethanol (i.e. 3% v/v or above) can lead to marked cell deterioration and is not desirable.

To further explore the ethanol related mechanism in which RBCs can be protected from INFeD induced damage, additional experiments with three healthy blood samples (Subjects P–R) were performed and ethanol was only administered 2 h after 6 h INFeD loading (Fig.5). Cell deformability restoration was observed with 0.03% ethanol treatment (subjects P and Q), which is very similar to that of ethanol co-administration experiments (p < 0.05). High ethanol concentration would reversely lower RBC deformability. The result suggests appropriate amount of ethanol not only protects but also restores RBC from INFeD induced mechanical damage. In addition, confocal laser scanning microscope (CLSM) imaging (Fig. 6) was also presented to show the change of erythrocyte membrane skeleton. The results indicated that the distribution of actin on membrane skeleton of RBCs with INFeD was discrete and the actin duplex is uncoiled. low concentration of ethanol administration (<0.3% v/v) after 6 h INFeD loading could partly recovered the actin of RBCs membrane skeleton and the content of actin was increased and evenly distributed in RBCs. However, 3% v/v ethanol brought about a lack of the actin resulting in an incomplete ring.

To further investigate the effect of ethanol on RBCs deformability, only ethanol with different concentrations was used to treat the RBCs at 37 °C for 2 h. The effects appeared to be subject-dependent and very diverse (Fig. 7). At 3% v/v ethanol loading, in two subjects (T and V), a significant increase in RBC deformability was detected (p < 0.05, Tukey test). Sonmez *et al.*^8^ have reported the similar result. To note, in subject T, increase in RBC deformability occurred at slightly higher ethanol concentration of 0.3% (p < 0.001, Tukey test). However, in two other subjects (subject S and U), not statistical significance can be concluded. It is definitely true that the observed effect is subject-dependent and complicated, and this is one of the reason why a rapid measurement of RBC-response to drug and ethanol would be useful in clinical setting, quickly determining potential of effect for a particular patient/subject.

## Discussion

*In vitro*, red blood cells cannot release iron from dextran. Iron chelated by dextran does not harm red blood cells to change the deformability. Therefore, the underlying mechanism of significant RBC stiffening at high concentration may be related to dextran. Dextrans specifically have been shown to adsorb to the RBC cell surface, whereas soft surfaces, such as the RBC glycocalyx, are characterized by a layer of attached macromolecules that can be penetrated in part or entirely by the free polymer in solution^15^. Goldsmith and Marlow’ have suggested that dextran may alter the mechanical or interfacial properties of the red cell membrane. But the cells in their study didn’t undergo extensional deformation^16^. Gerard B. Nash indicate that dextran has no effect on the elastic modulus and only a minimal influence on cell volume and surface area, but they found dextran induced a discocyte-stomatocyte shape transformation^17^, which has been discovered at higher INFeD concentration in our study. In addition, dextran can increase media osmotic pressures. The RBC deformability will decrease with increasing osmolality^18^. The differential dose-dependent deformability response in our study, therefore, is likely to be associated with individual’s cells susceptibility against INFeD overloading.

The possible mechanism accounting for the dose dependency of ethanol mediated cell damage is that at low concentration, alcohol molecules mainly situated on membrane surface, leading to an increase in lipids mobility^17^ and alcohol can activate the protein kinase A and protein kinase C of the RBCs with mechanical stress, accordingly improve the decreased filterability of RBCs. At higher concentration (3% v/v) however, alcohol molecules are pushed towards inner layers of the membrane, into the hydrophobic core, which then results in an expansion in the cytoskeleton^19^. Spectrin, an important cytoskeletal protein^20^ strongly associated with RBC morphology and deformability^21^, is located at the inner membrane of the red cells^22^. Previous study has reported that, with high concentration of ethanol radical, spectrin showed the highest loss among all other membrane protein^23^. Furthermore, studies also found that high concentration of ethanol could alter the osmolality of suspending medium, and thereby promotes the formation of echinocytosis (Fig. 4d)^9^. Additionally, at 3%, the destructive effect of ethanol could dominate: ethanol radicals was found to form covalent bonds with Hb^24^, which irreversibly alters the hemoglobin structure. Nevertheless, ethanol radicals and iron oxidation exhibit much milder destructive chemical effect on RBC Hb as compared to the hydroxyl radicals^1^, though all induce metHb formation. While the mechanistic study of RBC deformability is generally challenging, the ability to rapidly measure such changes in response to commonly used compounds would be able to provide clinically useful information, which is the main point we would like to get across in this paper.

To note, the dose-dependent changes in RBC deformability we have compared thus far are based on a “normalized value”, taking the values from control sample as normalized “1”. By doing so, we highlight the percentage change of cell deformability after INFeD loading, specific to individual donors. However, inevitably, this normalization procedure also overlooks inherent baseline deformability difference from different donors. We note a fairly wide range in the baseline RBC velocity with mean and standard deviation of 3.23 ± 1.09 units. This donor-dependent difference in RBC baseline deformability was also illustrated previously by comparing the red cell elongation index under constant shear stress^25^. We analyzed the changes in RBC velocity after 5000 μg/mL of INFeD loading as a function of their baseline velocity for all 18 subjects (subjects A–R). The INFeD induced changes of RBC deformability (Δv) exhibited a positive correlation with the initial or baseline RBC deformability (γ = 0.93, r^2^ = 0.86). A linear fitting with slope of 0.68 suggests that in average a 68% stiffening was observed for when subjects undergo INFeD loading.

## Conclusion

Overall, high concentration INFeD infusion was observed to reduce RBC deformability *in vitro* in a dose dependent, patient-specific manner. Significant variation in individual’s RBC deformability tolerance against INFeD was found and quantified. Besides, we also report, for the first time, that co-administration of ethanol, at a moderate dose, can partially alleviate INFeD-mediated RBC deformability damage. Meanwhile, we find ethanol can improve impaired cell deformability. This has important implications for patients with lower RBC deformability, such as thalassemia. Looking forward, the low-cost and on-site determination of individual RBC deformability with microfluidic technology could provide an additional dimension of clinically relevant information.

## Methods

### Sample preparation

Fresh human blood was centrifuged at 300 g for 3 min to remove the plasma and buffy coat. Packed RBCs were then added into RPMI 1640 medium (BioWhittaker RPMI-1640, Lonza) containing 20% of fetal bovine serum (Gibco by life technologies) such that final RBC solution contains 1% of packed RBCs. RBC solutions without INFeD or ethanol treatment was used as the control. In the INFeD loading experiments, RBC solutions were incubated with 0.5–5000 μg/mL of INFeD (Sigma) at 37 °C for 6 h. At the end of incubation, sample solutions were then washed with RPMI solution containing 20% FBS to remove excess INFeD in the medium. To prepare sample solution for the experiments requiring ethanol co-administration, 1% RBC solution with 5000 μg INFeD was first prepared and made into 4 aliquots. Ethanol was then added within 5 min so that final sample contained 0, 0.03, 0.3 and 3% v/v of ethanol. All samples were incubated at 37 °C for 6 h. For experiments treating ethanol after INFeD, RBC sample solutions containing 5000 μg/mL of INFeD were first incubated at 37 °C for 6 h. At the end of incubation, all samples were washed 3 times with RPMI medium containing 20% FBS at 300 g for 5 min to ensure any excess INFeD in the solution is removed. Different concentrations of ethanol were then added to respective samples and incubate at 37 °C for another 2 h. For all experimental conditions, at the end of the procedure, all samples were washed 3 times to remove excess INFeD or/and ethanol. RBCs were then resuspend with RPMI medium containing 20% FBS. Each RBC samples were divided into two parts, for deformability and metHb measurements. Sample solutions were adjusted with RPMI medium containing 20% FBS such that the final hematocrit is 0.2–1% for all. Cell tracker orange (Invitrogen) standard staining protocol was followed for improved image visualization. Fresh human blood samples were purchased from research blood components, LLC (http://researchbloodcomponents.com/). The study protocol was approved by American Association of Blood Banks. The methods were carried out in accordance with the approved guidelines. An IRB approved consent form is obtained from each donor.

### Deformability measurement

**Figure 1:**
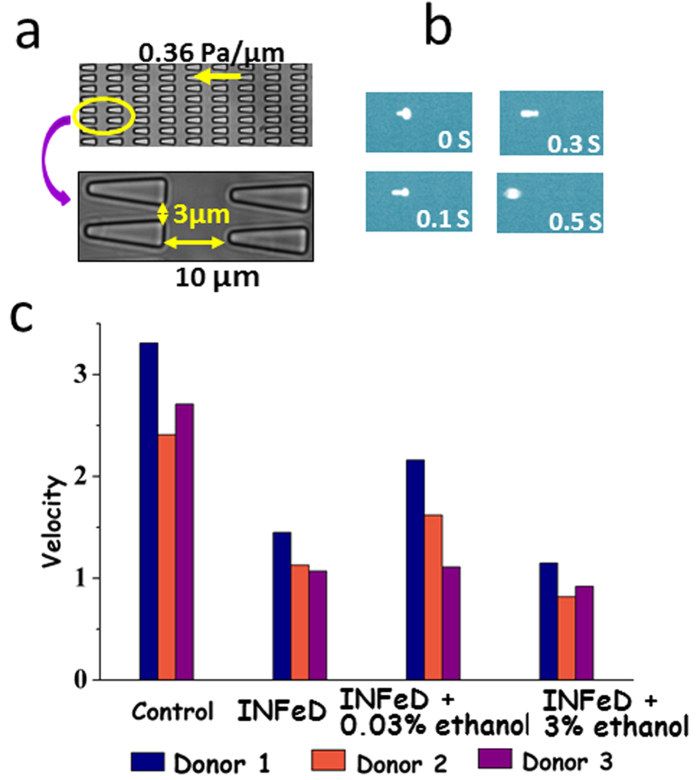
Experimental schematics. (**a**) The PDMS chip consists of repeated pillar array structures and main channels with triangular pillar arrays. The microfluidic device was driven by a constant pressure gradient across (0.36 Pa/μm). (**b**) Experimental images of RBCs traveling in channels. (**c**) RBC deformability: control, without any treatment; INFeD, only 5000 μg INFeD treatment ; INFeD + 0.03% ethanol, with 5000 μg INFeD and 0.03% ethanol treatment; INFeD + 3% ethanol, with 5000 μg INFeD and 3% ethanol treatment. INFeD and ethanol were added to the RPMI solution containing 20% FBS with 1% hematocrit. All samples were incubated at 37 °C for 6 h.

**Figure 2:**
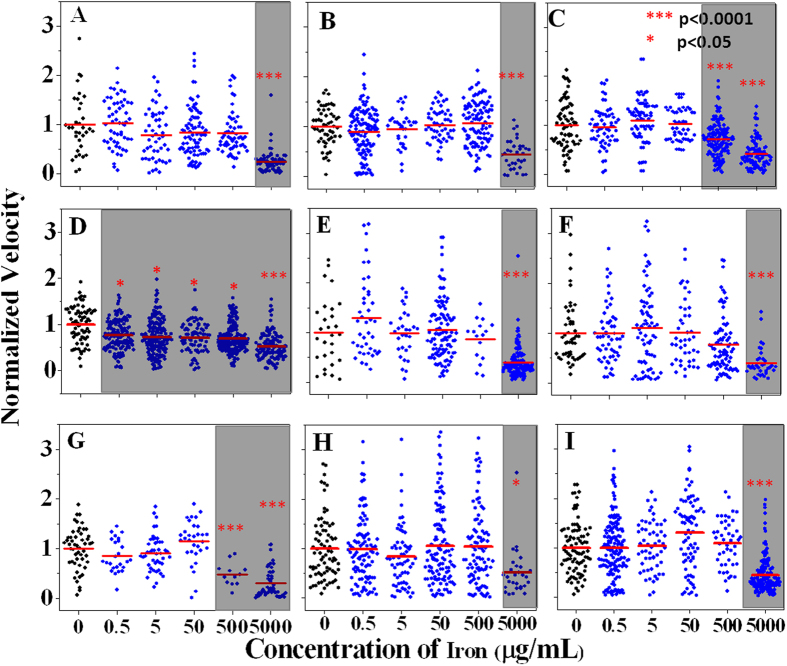
INFeD dose dependent changes in RBC deformability in all 9 subjects (**A**–**I**). Each dot represents a cell. The grayed areas are the INFeD concentrations which induce the RBCs deformability significant difference compare to control deformability. Velocity measurement is normalized against the average RBC velocity in the control sample. The samples with different concentration INFeD were incubated at 37 °C for 6 h.

**Figure 3:**
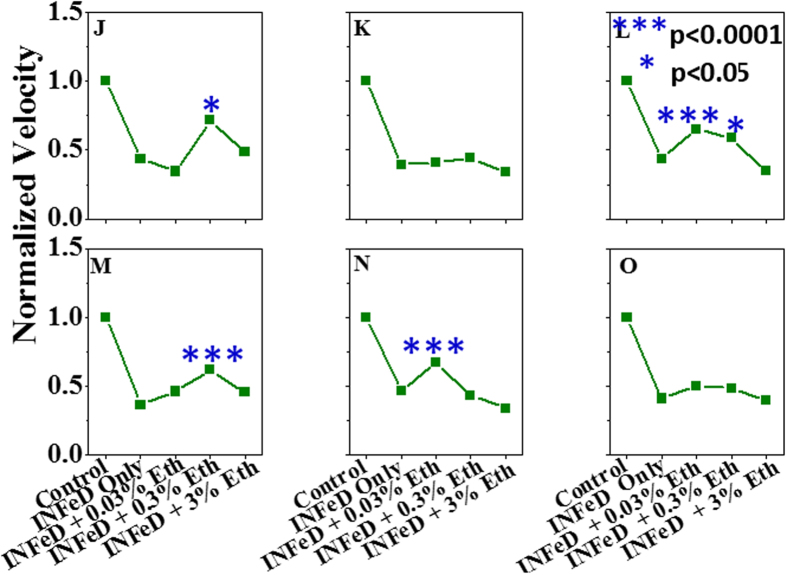
RBC deformability are measured to investigate the effect of ethanol co-administration. 6 subjects (**J**–**O**) were assessed for this effect. The sample treament method is same as Fig. 1c. The blue star denoted the significant difference of RBCs deformability of ethanol with 5000 μg/mL INFeD treatment samples compare to 5000 μg/mL INFeD treatment samples only.

**Figure 4:**
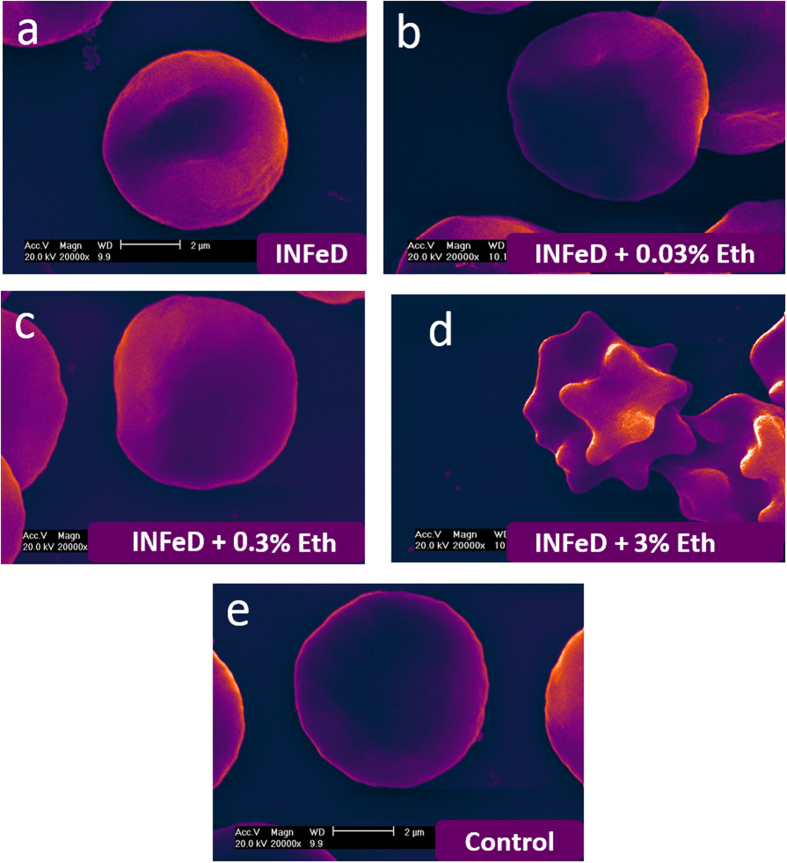
SEM imagings of RBCs with 5000 μg/mL INFeD only (**a**), 5000 μg/mL INFeD with different concentrations of ethanol (**b**–**d**) and with no treatment at all (**e**), Eth: ethanol.

**Figure 5:**
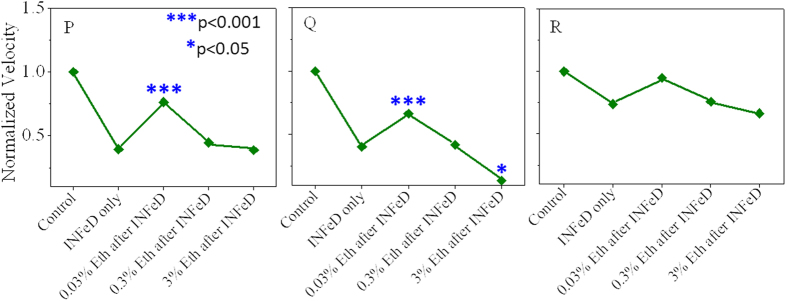
The effect of ethanol on RBC deformability after INFeD (subjects (**P**–**R**)). RBC sample solutions containing 5000 μg/mL of INFeD were first incubated at 37 °C for 6 h, and then excess INFeD in the solution was removed. Different concentrations of ethanol were added to respective samples and incubate at 37 °C for another 2 h. The blue star denoted the significant difference of RBCs deformability of ethanol treatment samples after 5000 μg/mL INFeD compare to 5000 μg/mL INFeD treatment samples only.

**Figure 6:**
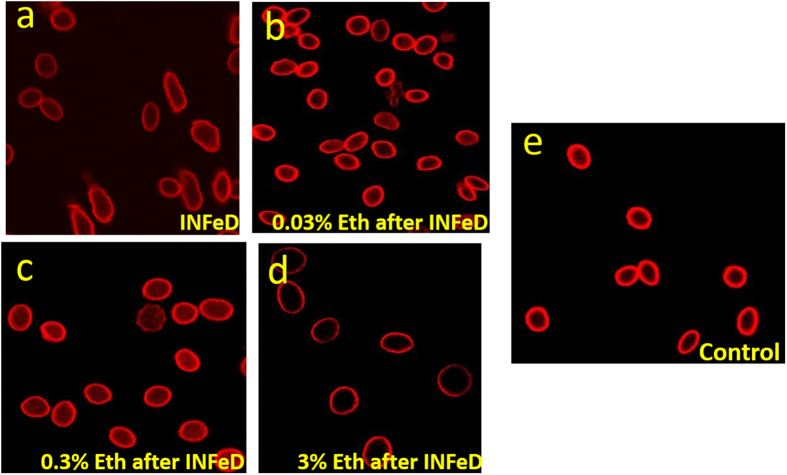
CLSM imaging of of erythrocyte membrane skeleton with 5000 μg/mL INFeD only (**a**), different concentrations of ethanol after 5000 μg/mL INFeD (**b**–**d**), and no treatment at all (**e**).

**Figure 7:**
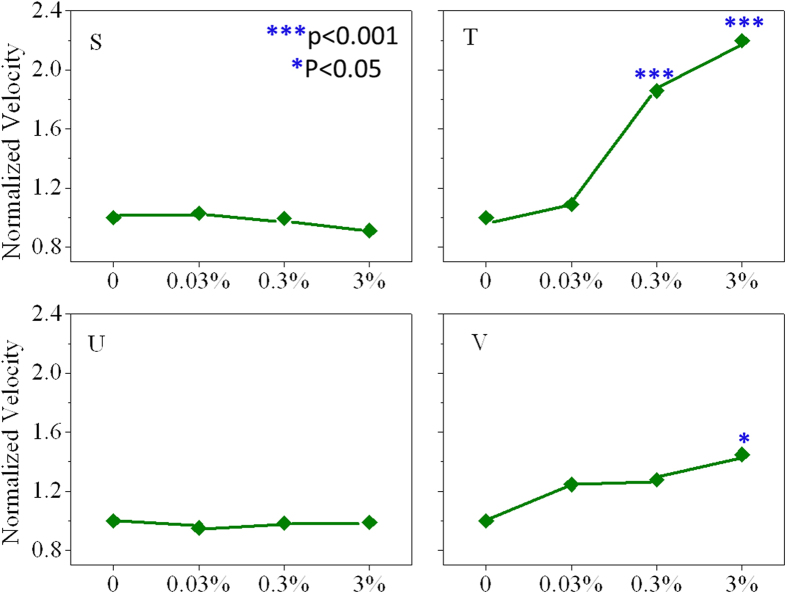
The effect of only ethanol on RBC deformability (subjects (**S**–**V**)). 1% RBC solution was made into 4 aliquots containing 0, 0.03, 0.3 and 3% v/v of ethanol,respectively. All samples were incubated at 37 °C for 2 h. The blue star indicated the significant difference of RBCs deformability of ethanol treatment samples compare to control.

The microfluidic device was designed with layout program and it consists of 500 μm × 500 μm inlet/outlet reservoirs and parallel capillary channels with triangular pillar arrays (Fig. 1a). Deformability device was mounted to the microscope (Olympus IX51, Center Valley, PA) connected to a CCD camera (Hamamatsu Model C4742-80-12AG). Approximately 3 μl of blood samples were loaded to reservoir of the microfluidic device. The inlet reservoir was connected to a vertically held 60-ml syringe which was partially filled with with RPMI medium containing 20% FBS buffer solution. A laminar flow was induced by gravity^11^,^13^,^26^. Images were automatically obtained by IPLab (Scanalytics, Rockville, MD) at 100 ms time interval and the post-imaging analysis was done using imageJ. Cell deformability is defined by the transit velocity of cells flowing in the microchannels. The velocity of individual RBCs was simulated with the following equation:


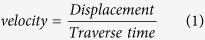


where displacement is the distance the cells moved; the traverse time is in seconds. Normalized velocity was calculated by dividing the velocity of individual RBCs from various experimental conditions by the control group RBC velocity on the same experimental day.

### RBC morphology imaging

RBCs were fixed with 0.1 M PBS containing 2.5% glutaraldehyde for 1 h, dehydrated with alcohol (30%, 50%, 70%, 90%, and 100%) and dried on the formvar membrane. All treatments were performed at room temperature. The cells were then coated with gold and placed into the Scanning Electronic Microscope (SEM) chamber. SEM images were obtained using an electron microscope with a voltage of 20 kV. (Philip XL30, FEI Company, Eindhoven, The Netherlands).

### CLSM imaging of of erythrocyte membrane skeleton

RBCs were prepared according to the experimental protocol of fluorescent phallotoxins instruction (Cytoskeleton). In short, fix the sample in 3.7% formaldehyde solution in PBS over night at room temperature, and then wash three times with PBS. Resuspend the fixed RBCs in 0.1% Triton X-100 in PBS for 5 minutes. After wash three times with PBS, add 10 uL of Rhodamine-Phalloidin (500 ug/mL of methanolic stock solution) on cells and incubate for 60 minutes at 4 °C. Rapidly wash three times with PBS. Place each coverslip and view using CLSM (Olympus FV10i).

## Additional Information

**How to cite this article**: Liu, L. *et al.* Study of individual erythrocyte deformability susceptibility to INFeD and ethanol using a microfluidic chip. *Sci. Rep.* **6**, 22929; doi: 10.1038/srep22929 (2016).
